# Insights into the diagnosis and management of congenital hypothyroidism

**DOI:** 10.1186/1687-9856-2015-S1-O18

**Published:** 2015-04-28

**Authors:** Paul Hofman

**Affiliations:** 1 Liggins Institute, Auckland, New Zealand

## 

Congenital Hypothyroidism is the commonest preventable cause of intellectual disability. Newborn screening with early identification and treatment has resulted in near normalisation of long term intellectual outcomes in the affected children. However, a number of issues still remain to be clarified. Firstly, while there has been a marked improvement in intellectual outcomes many studies have shown persistent cognitive deficits compared to control populations. It has generally been considered that this deficit reflected antenatal reductions in fetal thyroxine exposure and was unavoidable. However, variables such treatment delay and low thyroxine doses when initiating may also contribute. There is now data to suggest early diagnosis and treatment with high dose thyroxine and frequent blood testing can result in completely normal cognitive outcome when compared to healthy siblings. Treatment paradigms will be discussed that normalise cognition, with suggestions of management regimes that can be used in areas of the Asia-Pacific region where thyroid function testing is more difficult.

The frequency of congenital hypothyroidism is increasing world-wide. This is in part related to changes in the newborn cut-offs which have reduced in many countries. Other reasons have also been suggested and the ethnic composition of the countries seems very relevant. Asian and Pacific peoples appear to have a much higher incidence of dyshormonogenesis and rates are of congenital hypothyroidism are climbing more rapidly in countries where the proportion of these ethnicities is increasing.

There also remains ongoing concern about the appropriate cut-offs for notification of hypothyroidism. Indeed there has been an international trend for lower cut-offs resulting in many children being diagnosed with sub-clinical hypothyroidism. The relevance of diagnosing these children remains unclear with most likely being normal. I will discuss the use of different cut-offs and how these can affect the diagnosis and management of congenital hypothyroidism, as well as what should be the appropriate cut-offs in less affluent countries and the common reasons for missed diagnoses.

